# A Global Analysis of Enzyme Compartmentalization to Glycosomes

**DOI:** 10.3390/pathogens9040281

**Published:** 2020-04-12

**Authors:** Hina Durrani, Marshall Hampton, Jon N. Rumbley, Sara L. Zimmer

**Affiliations:** 1 Department of Biomedical Sciences, University of Minnesota Medical School, Duluth Campus, Duluth, MN 55812, USA; durra018@d.umn.edu; 2 Mathematics & Statistics Department, University of Minnesota Duluth, Duluth, MN 55812, USA; mhampton@d.umn.edu; 3 College of Pharmacy, University of Minnesota, Duluth Campus, Duluth, MN 55812, USA; jrumbley@d.umn.edu

**Keywords:** evolution, kinetoplastid, organelle, metabolic pathway, glycolysis, gluconeogenesis, meta-analysis, peroxisome targeting sequence, PTS1, PTS2

## Abstract

In kinetoplastids, the first seven steps of glycolysis are compartmentalized into a glycosome along with parts of other metabolic pathways. This organelle shares a common ancestor with the better-understood eukaryotic peroxisome. Much of our understanding of the emergence, evolution, and maintenance of glycosomes is limited to explorations of the dixenous parasites, including the enzymatic contents of the organelle. Our objective was to determine the extent that we could leverage existing studies in model kinetoplastids to determine the composition of glycosomes in species lacking evidence of experimental localization. These include diverse monoxenous species and dixenous species with very different hosts. For many of these, genome or transcriptome sequences are available. Our approach initiated with a meta-analysis of existing studies to generate a subset of enzymes with highest evidence of glycosome localization. From this dataset we extracted the best possible glycosome signal peptide identification scheme for in silico identification of glycosomal proteins from any kinetoplastid species. Validation suggested that a high glycosome localization score from our algorithm would be indicative of a glycosomal protein. We found that while metabolic pathways were consistently represented across kinetoplastids, individual proteins within those pathways may not universally exhibit evidence of glycosome localization.

## 1. Introduction

An accurate understanding of eukaryotic biology requires representation of studies from the widest possible spectrum of organisms. For instance, within the phylum Euglenozoa, studies of species of the order Trypanosomatida continually reveal new pathways and regulatory mechanisms that change what we believe to be true of the characteristics of eukaryotic organisms (e.g., new post-translational protein modifications [1], polycistronic transcription of eukaryotic nuclear genomes [2,3], genome-scale mRNA trans-splicing [4], and RNA editing [5]). The parasitic protozoan Trypanosomatida species that are insect transmitted and cause diseases in mammalian hosts are the best studied. However, comparisons between these species and their neighbors would be very useful in order to better understand the evolution of novel pathways and events within eukaryotic biology. These comparisons include those between the dixenous, well-studied Trypanosomatida species and others of the same order, many of which are monoxenous and/or have different hosts. Additional useful comparisons are between different orders of the class Kinetoplastea, of which Trypanosomatida belongs, including Parabodonida and the free-living Eubodonida [6], and between classes of Diplonemea, Euglenoidea, and Kinetoplastea of the phylum Euglenozoa.

One feature common to eukaryotes is the partitioning of enzymatic pathways and material into membrane-bound compartments or organelles. Some of these, such as a nucleus, are common to all eukaryotes. Others are unique to a subset of eukaryotic organisms. In Kinetoplastea and Diplonema, the first seven steps of glycolysis are compartmentalized into an organelle called the glycosome along with parts of other metabolic pathways [7]. The glycosome originates from the same common eukaryotic ancestor as peroxisomes, organelles found in a wider range of eukaryotes with which it shares similar biogenesis and import machinery [8]. Interestingly, while Kinetoplastea and Diplonema possess glycosomes (as defined by their inclusion of glycolytic enzyme content) but not peroxisomes, the opposite is true for the closely related Euglenoidea [9].

Our understanding of the complement of glycosome-localized metabolic enzymes is centered on experiments cataloguing them in *Trypanosoma brucei*, and to a lesser extent *Trypanosoma cruzi* and *Leishmania* spp.—all dixenous organisms capable of causing human and livestock disease (e.g., [10,11,12]). As the *Trypanosoma* and *Leishmania* genera are phylogenetically distinct, the fact of apparent overlap in their glycosome enzyme composition suggests a high degree of conservation [13]. However, pioneering studies in the kinetoplastid relative Diplonema [14,15] suggest that beyond the seven common glycolytic enzymes, their glycosomes lack other enzymes that are glycosomal in the well-studied kinetoplastid species. Our question is whether evolution and maintenance of glycosome contents may be influenced by organism life cycle and lifestyle as much or more than phylogeny, which would require probing their composition in monoxenous and free-living species, and in dixenous organisms with hosts other than mammals. The answer to this question is confounded by the probability of dual localization of enzymes [12], duplication and separate localization of paralogues (e.g., [16,17]), and the reality of the intracellular connectedness of peroxisomes with other membrane-bound compartments that muddies the waters of subcellular fractionation experiments [18].

Our study objective was to determine the extent that we could leverage existing experimental studies in model kinetoplastids to determine the composition of glycosomes in species for which no experimental evidence is available. This objective required a better in silico identification scheme for signals within predicted proteins that could classify their localization as likely glycosomal. We anticipated that signal-to-noise ratio would still be a challenge in our output. However, enough laboratory-based glycosome composition studies in three species plus enough kinetoplastid sequenced genomes are now available to make our intermediate-throughput analysis possible. Our final work demonstrated that an iterative approach combining automated analysis with manual protein evaluation and annotation can be beneficial in uncovering localization patterns of glycosome proteins among the kinetoplastids.

## 2. Results

### 2.1. Meta-Analysis of Existing Studies

**Figure 1:**
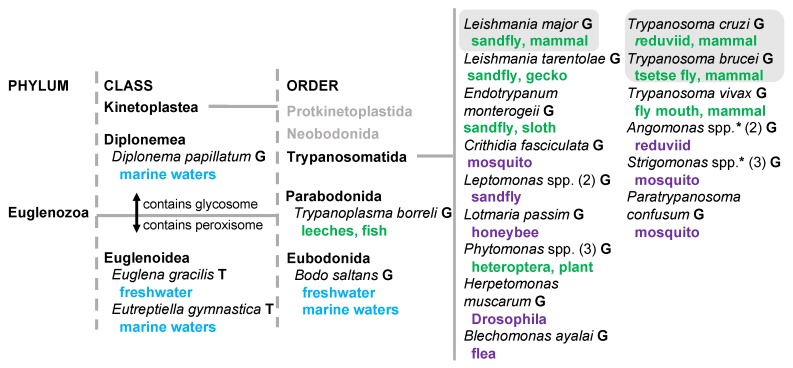
Lifestyle of Euglenozoa species for which glycosome targeting was analyzed. Hosts or environments for these species are shown in color. Blue indicates water environments of free-living species, green indicates hosts of a dixenous lifestyle, and purple indicates insect hosts of a monoxenous lifestyle. For some genera, number of species analyzed is included in brackets. Species included in the initial meta-analysis of glycosome localization are compartmentalized in a grey background box. G, genomic DNA was analyzed. T, translated transcriptomic data was analyzed. Asterisk indicates genus with bacterial symbiont.

In order to establish a starting population of proteins for which glycosomal localization is well supported by experimental evidence, we performed a meta-analysis that included as many different information types as possible. We included studies in species *Trypanosoma cruzi* and *Leishmania donovani* in addition to *T. brucei* where most work is performed (Figure 1, [10,11,12,19,20,21,22]). These three species, all dixenous mammalian parasites, are still similar in lifestyle relative to the full complement of species containing glycosomes. The studies performed in these species utilized a variety of methods. The methodologies for establishing localization or organelle content on a global scale have strengths and weaknesses. Isolation of purified organelles followed by mass spectrometry has revolutionized our understanding of the protein composition of subcellular compartments and structures. However, it is important to remember that proteins will not be equally identifiable by mass spectrometry, as some lack ideal cleavage sites and positions for liberation of peptides of appropriate size and composition, and sample preparation will favor the identification of some proteins over others [23]. Conversely, methods involving fluorescent tagging of proteins followed by microscopy are problematic if tags on the N- or C-terminus effectively block terminal transit peptides and localization signals. There is no perfect single method for this sort of analysis, thus combining results of studies using different methodologies can be useful.

We incorporated data of three types. Most studies involved glycosome purification followed by protein composition analysis by mass spectrometry or other methods. When necessary for these studies, we took protein datasets prior to any culling by prediction of peroxisome targeting sequences (PTS1 and PTS2, see below) that the authors may have performed. The second type of data came from the TrypTag project in which *T. brucei* proteins are endogenously tagged with GFP (Green Fluorescent Protein) at one of their native loci [21,24]. Microscopy images of the TrypTag GFP localization pattern within the cell were individually analyzed for each protein for which TrypTag tagging had been successful at its N-terminus, and compared with the distinctive visual pattern of multiple oval-shaped glycosomes shown and described in [24]. The final source of data incorporated was a protein’s presence in the resource PeroxisomeDB [19] for any trypanosomatid species. Peroxisome DB is no longer updated, as entries for literature utilized for the resource cease prior to 2010. We treated this as an “archival” source of glycosome localization evidence based off of early individual protein studies in which the protein was determined to be glycosomal.

After inclusion of proteins from all sources, 302 proteins were determined to be glycosomal in at least one study. We subtracted 92 as being part of the import/export machinery or part of the protein composition of the glycosome membrane, as we were instead focused on proteins found in the matrix of the glycosome. We eliminated duplicates when observed. The remaining 209 proteins were then considered as possible glycosome matrix proteins (Table S1). This list consists of 188 that are likely metabolic enzymes and 21 that are hypothetical proteins with no discernable identifying motifs according to TriTrypDB [25]. We also were unable to find any motifs with common motif-finders.

We ranked these enzymes in order of reliability of the data that suggests glycosomal localization. We utilized Bayesian-guided data reliability weight assignments. A glycosomal signal pattern of a tagged protein received a high weight, while a non-glycosomal fluorescence pattern, being less reliable, received a lesser negative weight. Protein studies received a range of individual rankings (Materials and Methods). It is certainly true that another weighting scheme could have resulted in the inclusion or exclusion of lower-ranking proteins from our final group; however, most of these proteins would be retained in our final collection regardless of alternative weight assignment to various studies.

We decided on a cut-off weight (a value of 5) that, except for 1 enzyme (Hypothetical protein Q38C56), exhibited evidence in more than one species. The total number of proteins in our Glycosome Conserved Enzyme Collection (GCEC) is 57 (Table 1). Not surprisingly, the seven first steps of the glycolytic pathway (including triose phosphate isomerase) were clustered within the first 28 highest proteins scored for reliability of glycosome localization. Other known glycosomal pathways were also well-represented such as the pentose phosphate pathway, purine metabolism, and pathways shared with peroxisomes such as fatty acid metabolism. Clearly, as over a hundred proteins are typically identified as potentially glycosomal in any single proteomic study, this list is not comprehensive. Furthermore, individual studies have characterized glycosomal proteins that are not in GCEC. For instance, it is explicitly shown that several superoxide dismutase enzymes are glycosomal from individual *T. brucei* studies, yet this protein does not appear extensively in the global studies of our meta-analysis, so that it is not included by our methods [16,17]. Interestingly, 8 proteins (14%) with sufficient evidence to appear on the list are hypothetical (Table 1). The systematic approach to localization provided by TrypTag has been helpful in bringing these proteins to light, as the fluorescence pattern of their tags all indicated glycosomal localization. TrypTag localization was the only evidence for glycosome localization in six proteins in the master Table S1. In addition to two that are hypothetical, the others are an aspartate carbamoyltransferase that, while found soluble in *Leishmania* in 1981, may be glycosomal in *T. brucei*, as it is part of a pyrimidine biosynthesis pathway with other glycosome components [26]. Others are a ribonucleoside-diphosphate reductase (purine biosynthesis), a mannosyltransferase that likely participates in glycosylphosphatidylinositol-anchor biosynthesis (possibly appearing localized to glycosomes based on the ER’s glycosome associations), and a protein with putative phosphatase activity. Other phosphatases have been identified as glycosomal [27].

After establishing the enzymes that would be part of the GCEC, we categorized them into major Kyoto Encyclopedia of Genes and Genomes (KEGG) pathways by assigning them KEGG designations (Table 1). Our goal was to limit the number of pathway categories to allow for patterns to emerge, yet avoid the level of category breadth that erases the essential metabolic depth. Kinetoplastid UniProt accession numbers were used to retrieve primary KEGG reference orthologs and putative pathway associations. Pathways retrieved were organized top to bottom, highest to lowest specificity for any given enzyme. Generally, the top metabolic pathway was retained for our purposes but in some cases more specific subclasses were used, (i.e., urea cycle). Ultimately, nine major KEGG pathways were identified that were represented by two or more enzymes and eight pathways were represented by only one enzyme. Proteins deemed hypothetical could not be categorized due to lack of orthologous sequences.

### 2.2. An Adequate Algorithm for Glycosome Localization

It is well understood that many peroxisome proteins possess either a peroxisome targeting signal (PTS1), which is the amino acid triplet [Serine-Lysine-Leucine] on their C-termini, or less commonly, the less-conserved PTS2 very near their N-termini. The predictive power of the PTS1 signal for glycosome localization was analyzed in the most stringent and comprehensive *T. brucei* glycosome proteome study. The conclusion was that the PTS1 signal had a sensitivity of less than 40% and a specificity of less than 50%, making it a remarkably poor predictor of localization [12]. One potential reason for this is that the calculation did not differentiate glycosome membrane and surface proteins from matrix proteins. At least some membrane proteins of peroxisomes originate in the ER [28], and thus utilize an entirely different localization mechanism than the compartmentalized matrix enzymes. A second reason is that presumably only the canonical PTS1 signal, ‘SKL’, was used in the analysis. It is likely that in kinetoplastids, as in plants [29], certain conserved variations on the canonical sequence are also functional targeting signals. 

**Figure 2:**
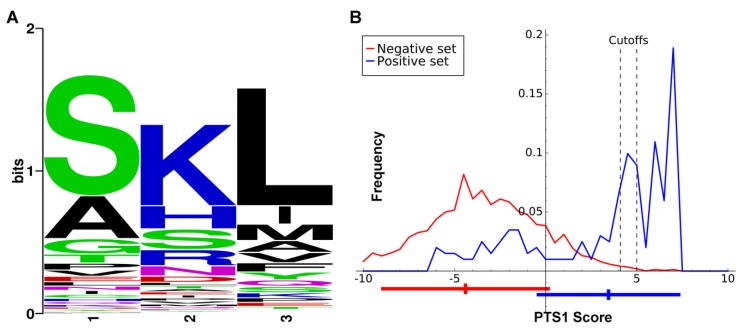
(**A**). The amino acid bias of the C-terminus of proteins in our Glycosome Conserved Enzyme Collection (GCEC) training dataset from *Trypanosoma cruzi*, *Trypanosoma brucei*, and *Leishmania donovani* (*L. major* homologues of *L. donovani* proteins were used in the amino acid bias calculations). The last three amino acids of the proteins could harbor glycosome targeting signals (glycosome PTS1s). The position of the first amino acid of the PTS1 is position 1, with the final amino acid of the protein being position 3. (**B**). Frequencies of PTS1 scores for GCEC proteins (blue, positive set) and a dataset of non-glycosomal proteins (red, negative set). Bars shown underneath the graph indicate mean and standard deviation of each dataset. Cutoff values are shown as vertical dotted lines for pre-selected protein datasets (left) and whole genome prediction (right).

A major motivation for setting the evidence bar very high for a protein to be included in the GCEC dataset is that we subsequently used it to determine whether there were features of glycosome signaling peptides that differed from the peroxisome targeting signals previously used to interrogate collections of glycosome proteins [13]. The number of proteins utilized is similar to that used to train the plant PTS1 prediction tool PredPlantPTS1 [30], as plant peroxisome proteins can possess PTS1 sequences that are certain conservative substitutions of the consensus ‘SKL’. Our approach was similar to that used to generate more recently derived plant PTS1 prediction algorithms [29]. If the kinetoplastid glycosome targeting signal is similar to the PTS1 of plants, any length of input data from three to 40 amino acids will yield similar results. The positive training dataset was the C-terminal 20 amino acids of the GCEC training dataset (using the orthologues from all three species). The negative training dataset consisted of the remainder of the 35,640 proteins in the UniProt database from the same species. After initial comparison, we concluded that only the last three amino acids would be utilized in our glycosome-specific PTS1 predictor, for which amino acid composition preferences were marked (Figure 2A). These amino acid composition preferences were used to establish the algorithm for our PTS1 predictor for glycosomal proteins, which returns a numerical value from 7.35 (classical PTS1 ‘SKL’ sequence; high likelihood of glycosome localization) to −23.9 (File S1). Cumulatively, the PTS1 proteins returned a mean score of 3.45 ± 3.85. For the negative dataset, the mean score is −4.40 ± 4.54. Figure 2B presents the score distributions.

Predication software cutoff values for classifying the last three amino acids of a protein as a glycosome-specific PTS1 are user-selected. The cutoff must account for the tradeoff between specificity and sensitivity, with a higher cutoff value (maximizing specificity) being more appropriate for whole genome prediction, and a lower cutoff value (minimizing false negative rates) more suitable for examining localization of pre-selected protein sets [29]. In this work, we selected a cutoff value of >4.1 for examination of our evidence-based list of proteins (Table S1) and 5 for perusal of complete genome datasets. We based the value of 4.1 on the inflection point of the GCEC training set protein curve after which score frequency sharply increased, and the value of 5 on the point at which the frequency of the negative dataset approached zero in Figure 2B. While a prediction program for a second, glycosome-specific N-terminal signal analogous to PTS2 was desired, only three proteins in the entire dataset had a “classical” PTS2; therefore, this dataset was not deemed appropriate for development of such a tool.

### 2.3. Glycosome Localization Prediction of Orthologues of Gcec Proteins across Kinetoplastids

**Figure 3:**
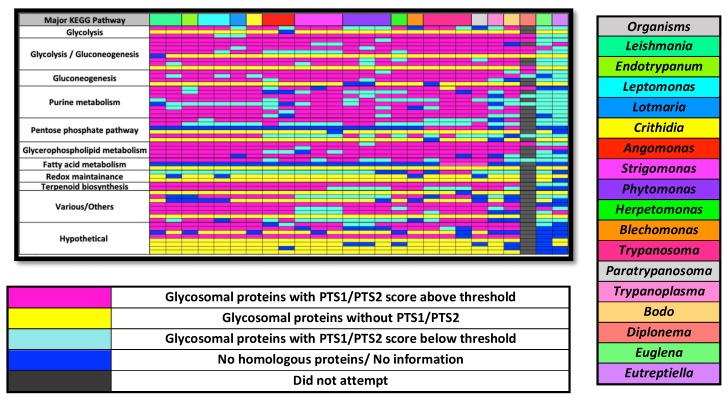
Map of glycosome targeting signal conservation across kinetoplastids, select euglinids and *Diplonema papillatum*. Different species and isolates of a genus are given separate columns. Each row represents one protein. Black rectangles in the *D. papillatum* column represent proteins for which we did not attempt to find an orthologue. The proteins in yellow are those that across all kinetoplastids do not possess a PTS1 or conserved PTS2. The *D. papillatum* entries that were used were those identified in [15]. Specific putative proteins and orthologue Uniprot/TriTryp/NCBI/contig numbers represented by each rectangle are found in Table S2. Organism columns are ordered loosely on phylogeny.

Both Leishmania and Trypanosoma species are represented by studies in our global analysis and showed compositional overlap, so similar to earlier conjectures [13], we hypothesized that the contents of the glycosomes are largely conserved between kinetoplastid species. Prediction of localization by presence or absence of a signaling peptide will suggest whether that hypothesis is more or less likely to be true. In order to utilize the glycosome-specific PTS1 prediction algorithm on kinetoplastids that are not part of the major human infectious trypanosomatids, we collected homologues of the 57 GCEC proteins in available kinetoplastid genomes or transcriptomes. When the genome was available in TriTryp [25], we accessed the genomes through the site’s BLAST portal as it allowed verification of synteny for the genes. As expected, gene duplications were present in the Leptomonas and Leishmania lineages prone to polyploidy. Some genes were missing entirely from specific genomes. This may result from genome sequencing or assembly issues, or the genes may in fact be missing from these species (Figure 3 blue boxes and Table S2). We assembled predicted homologues from all available kinetoplastid genomes/transcriptomes for each of the GCEC proteins into File S2. We stress that for species not represented in TriTryp, the protein of greatest sequence similarity to the *T. brucei* representative of each GCEC protein may not in fact be a true orthologue, but merely a protein with a conserved domain. This introduces potential but unavoidable noise into our analysis.

Predicted homologues and the training dataset were subject to PTS1 prediction using our previously developed algorithm, and PTS2 prediction using the expanded identification [HKQR][LVIFYA]{5}[HKQR][LVIFYA] [13] that started within the first 20 amino acids. Because of the low specificity of the PTS2 sequence, we only considered a protein with a PTS2 signal likely if the actual PTS2 domain was conserved in both sequence similarity and position, in that protein, in at least than 75% of the interrogated kinetoplastid species. Proteins with a PTS1 or conserved PTS2 are color-coded in pink in Figure 3, while proteins lacking such domains are colored cyan. 

The GCEC dataset also contained 16 proteins that contained no identifiable PTS1 or PTS2 across any of the GCEC organisms, and these were labeled yellow in Figure 2. In only one instance, triose phosphate isomerase, among all interrogated kinetoplastids and diplonema does an orthologue possess evidence of a signaling peptide where none existed in the GCEC organism enzymes. For this enzyme, a PTS1 is apparent in diplonema only. However, a potential cryptic PTS2 is present from approximate amino acids 48 to 55 in 17 of the 23 kinetoplastid species and isolates examined (cryptic because of its downstream position relative to the amino terminus). In the case of peroxisomes, a subset of proteins with very high experimental evidence of localization also lack identifiable targeting signals, and poorly understood or unknown import mechanisms are normally invoked for those proteins. It appears, then, that glycosomal proteins fall into two classes. The ones that have no identifiable signal among our GCEC species universally have no signal among kinetoplastids, while the others primarily do possess a targeting signal but it may be absent in orthologues of some species. We suggest that if an enzyme of the GCEC dataset is lacking a signal normally present, it may be excluded from the glycosomal contents and instead may be present in another cellular location. However, we cannot exclude the possibility that these proteins sporadically acquire the ability to “piggyback” on other proteins, or simultaneously acquire a yet-unidentified alternative localization signal to gain access to the glycosomal matrix. Two enzymes, orotidine-5-phosphate decarboxylase involved in pyrimidine metabolism, and mevalonate kinase of the terpernoid biosynthetic pathway, are the only ones for which evidence of glycosome localization is universally conserved across kinetoplastids.

For perspective on how random the event of a “lost” localization signal is among kinetoplastids, we included multiple species and/or isolates of genera when possible. Reassuringly, often (but not always) when a GCEC enzyme is missing a PTS1 or PTS2 within a genus, the loss is consistent across most or all orthologs of that genus (e.g., compare the loss of proteins across the three Phytomonas, three Strigomonas, or two Angomonas species/isolates in Figure 3). We especially noted the propensity of the PTS1 signal to not be present on homologues of pyruvate phosphate dikinase (glycolysis), ribokinase and 6-phosphoglucolactonase (pentose phosphate pathway), and putative thymine-7-hydrolase in species with a bacterial endosymbiont (Figure 1). This phenomenon suggests that some feature of organismal metabolism within the genus or group may be the driving force for alternative localization. Another validation of our approach to sorting out glycosomal localization by PTS1 or PTS2 is that the localization signals are largely lost in orthologous proteins of the Euglenoidea class that possesses peroxisomes rather than glycosomes (Figure 1 and Figure 3, last columns).

**Figure 4:**
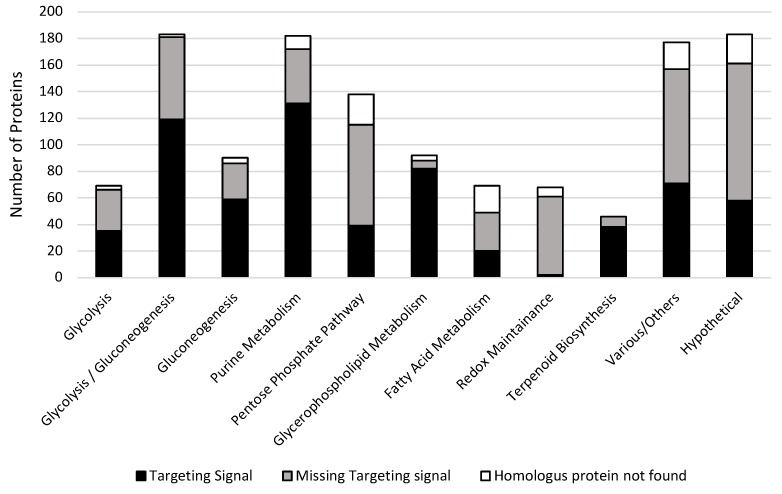
Major metabolic pathways of proteins of the Glycosome Conserved Enzyme Collection (GCEC). All orthologues of the proteins in each metabolic pathway that possess a glycosome-specific targeting signal 1 (PTS1) or a conserved peroxisome targeting signal 2 (PTS2) were classified as “Targeting Signal”; black. Orthologues in which the signal was either not retained, or throughout the orthologous group there was no evidence of either signal were both classified as “Missing Targeting signal”; grey. When an orthologuous protein was not found in a kinetoplastid, it was considered “Homologous protein not found”; white.

We note that in no KEGG pathway category does a single kinetoplastid species ever entirely lose representation within the glycosome signal-containing enzymes, even given that we are analyzing only a subset of glycosomal proteins. We asked whether proteins of certain metabolic pathways are more likely to conserve glycosome localization across the kinetoplastids than other pathways. Figure 4 tallies all of the orthologues of GCEC dataset enzymes into their respective KEGG pathway categories. One observed trend is some categories such as redox maintenance, pentose phosphate pathway, and the “hypothetical” proteins – those possessing no easily identifiable motifs – possess disproportionally more proteins that lack an identifiable PTS1 or PTS2 among all species. This is likely due to, in the case of the hypothetical proteins, a lack of traditional targeting signal across all orthologues of most hypothetical proteins. It is difficult to conjecture as to what this might mean. We also note that between KEGG pathway categories, there appear to be differences in the relative degree to which a glycosomal PTS1/PTS2 signal is conserved. For instance, a higher proportion of glycophospholipid metabolism enzymes conserve their signaling motifs relative to the proportion of pentose phosphate pathway and purine metabolism proteins that have their signaling motifs conserved. With this limited dataset, however, validation of these trends is likely not statistically possible. More proteins would be required to confirm that enzymes of specific KEGG pathway categories possess different degrees of overall localization with the glycosome. In summary, while we find that the metabolic pathways contributing enzymes to the glycosome are entirely conserved, we have evidence of variability of localization of specific enzymes among kinetoplastids.

### 2.4. Utilizing the PTS1 Signal Algorithm in Other Datasets

With the results of Figure 3 in hand, we next asked how many enzymes possessing some degree of experimental evidence of glycosome localization scoring below the top GCEC (Table S1) additionally had signal peptide evidence of glycosome localization. An enzyme was considered positive if at least one of the three species (*T. brucei*, *T. cruzi*, and *L. major*) had a glycosome-specific PTS1, or else if all three of the species had a PTS2 in which the motif began within the first 20 amino acids in order to capture potential contributions of sequences that are near neighbors to the final three amino acids. Before applying these standards, we removed twenty-two proteins that, visualized in the TrypTag collection, were obviously targeted to either the nucleus, flagella, mitochondria, or kinetoplastid. While there is a possibility that these proteins could be dual-localized, they also could be part of published glycosome proteomes because cellular compartments physically interact with each other [18] and perfect separation or purification of organelles is not possible.

Of the 126 remaining non-GCEC enzymes, 38 had targeting signals (Table 2), which constitutes 30% of the total. We then asked whether these additional enzymes could be classified with KEGG designations that were either already represented in our GCEC or else specific to peroxisomal pathways. This would constitute further evidence of glycosomal localization for these proteins. We used the same strategy for applying KEGG designations as for GCEC enzymes of Table 1, except that this time some enzymes could not be as precisely defined or lacked orthologues in well-studied systems and could be part of any number of KEGG pathways. Two of the 13 proteins deemed “hypothetical” within the interrogated 126 proteins again also appeared within the signal-peptide containing dataset. In all, 23 enzymes (60%) could be assigned to one of the major KEGG pathways that were represented among the GCEC proteins. Overall then, there is good evidence that the proteins in Table 2 are also localized to the glycosome, at least in the species in which localization signals are present.

Finally, we turned our attention to the question of how extensive the predictive power of the possession of a PTS1 is when interrogating genomes for likely glycosomal proteins. As only 62% of GCEC enzymes have a PTS1, clearly it cannot be used to identify the entire complement of glycosome proteins. However, we wanted to establish how likely it is that a protein found in a genome database with a PTS1 score above a certain level using our glycosome-specific algorithm is indeed targeted to the glycosome. We used the higher-stringency PTS1 cutoff score of 5 or greater to interrogate the dataset of the 81,604 Uniprot Kinetoplastid proteins. This dataset is biased towards organism genomes that previous investigators were interested in capturing, and thus includes the unusual mix of putative proteins of *Leishmania* spp., both lineages of the hybrid *T. cruzi* strain CL Brener, *T. brucei brucei*, *Trypanosoma theileri*, and *Bodo saltans*. However, it is convenient and representative of available datasets. 

Of those 81,604 Uniprot Kinetoplastid proteins, only 572 had a PTS1 score of 5 or more (Table S3). As there was no way to further evaluate the proteins that were hypothetical, they were removed. Four hundred twenty-six proteins possessing some identifiable motif remained that were manually evaluated: 202 were actually in our GCEC (Table 1) or were likely orthologues. This number increased to 287 proteins when including all proteins with some experimental evidence of glycosome localization (i.e., found in Table S1). Twenty-two entries had evidence of mitochondrial localization by virtue of the word “mitochondrial” in its name or else are a known mitochondrial protein or a homologue. In other organisms, a subset of mitochondrial protein mRNAs exhibit localization to ribosomes physically associated with mitochondria, and mitochondrial proteins synthesized in the cytosol are most likely highly associated with specific chaperone proteins [31]. It is reasonable to conjecture that in either of these contexts, a PTS1 may exist on a mitochondrial protein that may not be competent to deliver that protein to the glycosome. In conclusion, 76% of the proteins possessing a PTS1 score of 5 or higher had some other evidence of glycosomal localization, or its absence from Table S1 could be explained by a PTS1 overridden by mitochondrial localization (Table S3). 

One category of glycosomal proteins that was absent from the set with PTS1 values of 5 or higher were the peroxin (PEX) peroxisome protein import and biogenesis complex proteins. These proteins, best characterized in yeast, mammals, and plants, are loosely conserved in glycosomes and characterized to varying extents [32]. The highest-scoring peroxin of the Uniprot Kinetoplastid proteins was PEX13.1 with a PTS1 score of 4.3. This strengthens our theory that matrix proteins are more likely to require a PTS1 or PTS2 to be properly localized than glycosome membrane proteins or complexes that may derive from the likely glycosome biogenesis involving the ER or even the mitochondrion [28,33]. In the case of PEX13.1, the protein is dual localized, and its PTS1, “TKL”, is known to be important for its glucose-dependent glycosome localization [34]. We also analyzed the remaining ~100 proteins to determine how many possessed domains indicating a role in pathways clearly unrelated to glycosome function. (e.g., nucleic acid binding or processing; ribosome protein subunit). We estimate these proteins to comprise less than 25% of this protein list (highlighted in Table S3). This is a considerably better predictive outcome than was anticipated by the previously described assessment that for glycosomes, a PTS1 signal has a sensitivity of less than 40% and a specificity of less than 50% [12]. 

### 2.5. Similarities of Protein Compositions of the Glycosome with the Peroxisomes and the Mitochondrion

**Figure 5:**
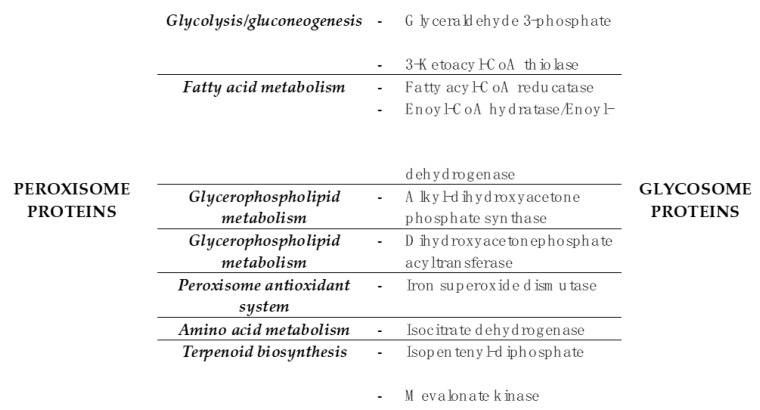
Overlap in glycosome and peroxisome metabolic pathway and enzyme composition. Proteins with experimental evidence of glycosome and peroxisome localization in the kinetoplastid and mammalian/plant systems, respectively, are arranged by metabolic pathway.

It is known that some basic metabolic pathways are conserved between peroxisomes and glycosomes, such as elements of fatty acid metabolism. We wished to generate a more specific picture of the enzyme conservation between these two organelles. We compared GCEC proteins and those with lower degrees of experimental evidence but that possess PTS1 or a conserved PTS2 within the first 20 amino acids (proteins of Table 1 and Table 2), to peroxisome metabolic enzymes from recent proteomic studies in mammal and plant [35,36]. Figure 5 shows the nine enzymes that are present in both organelles. The data suggest that particular enzymatic steps of fatty acid and glycerophospholipid metabolism and terpenoid biosynthesis, and superoxide dismutase activity may have been compartmentalized fairly early in the evolutionary history of the peroxisome/glycosome. This does not preclude the possibility that these activities are also present at other subcellular locations, as we know to be true of proteins of the pentose phosphate pathway, for example (evidence summarized in [37]).

Finally, we looked at the rather obvious overlap between metabolic pathways with components existing in both the glycosome and the mitochondrion. For instance, elements of fatty acid metabolism and enzymes involved in scavenging reactive oxygen species are found in both organelles. Therefore, it is possible that PTS1-containing proteins that we exclude from our analysis because of mitochondrial localization evidence may, in fact, dual-localize. However, many of the 22 proteins in the UniProt PTS1 > 5 population that we deemed mitochondrial are proteins of the electron transport chain localized to the mitochondrion membrane, or are related to electron transport. This pathway is not present in glycosomes, so we believe that other mechanisms override the strong PTS1 that would otherwise localize these proteins to the glycosome. Likewise, all of the proteins that we removed from the analysis resulting in Table 2 are proteins that, when tagged, exhibited mitochondrial localization patterns and/or were known mitochondrial proteins in at least one organism. Also, a possible feature of having a PTS2 is the potential for strong representation of the amino acid arginine. This amino acid is also over-represented in the beginning of many mitochondrial proteins [38,39]. Therefore, the degree of parallel or integrated processing [33] and/or dual localization is unresolved. 

## 3. Discussion

Environmental influences and organism lifestyle profoundly affect energetic flux through anabolic and catabolic metabolic pathways, and regulatory and protective processes. Examples of this can be found in the kinetoplastids, which include free living and monoxenous and dixenous parasites with a wide host range that is likely still incompletely defined [6]. For instance, the absence of the protective enzyme catalase in dixenous but not monoxenous kinetoplastid genomes may be a result of a requirement for low levels of the differentiation signal hydrogen peroxide for the dixenous organisms [40]. 

Most analysis of glycosomes has occurred in the dixenous *T. brucei*. The importance of glycolysis to *T. brucei* survival is life stage-dependent. Glycosome matrix enzyme composition of cells of the life stage replicating in the amino acid-rich tsetse fly gut differs substantially from those of the replicating life stage in the glucose-rich mammalian bloodstream, a concept nicely illustrated by 2D electrophoresis of purified glycosome lysate in 1990 [20]. This change in response to available nutrients is hypothesized to be the major environmental pressure that maintains a glycosome that contains metabolic as well as oxidative enzymes. Efficiency in establishment of, and changes to, gene product abundances is advantageous. In *T. brucei*, it appears that during changes in nutrient availability and life stage, selective turnover of entire glycosomes occurs. Concurrently, glycosomes containing a different enzyme composition are generated [41,42]. Glycolytic compartmentalization presumably allows *T. brucei* to utilize this process to achieve rapid adaptation.

A complicating factor for this explanation of why glycosomal contents are maintained throughout the kinetoplastid lineage is the fact that the metabolic remodeling of *T. brucei* is very extreme between its replicative stages in its mammalian and insect hosts [43]. Metabolic studies demonstrate that dixenous mammalian kinetoplastids that replicate intracellularly, rather than in the bloodstream as *T. brucei* does, do not have as radical a metabolic transformation, [43]. Even *T. brucei* may experience alternative metabolic states when in fatty tissues rather than in the blood [44]. Furthermore, it is more difficult to envision that the capacity for rapid metabolic adaptation is as much an evolutionary force for organisms that spend their entire existence in a single life stage, a single host, or in an environment such as saltwater. Finally, pathways such as purine metabolism are also represented in the glycosome. What is the reason for retaining compartmentalization of these pathways across kinetoplastids?

Our findings serve as building blocks from which we can begin addressing questions of the origins and maintenance of glycosomes. We have obtained a collection (the consolidation of Table 1 and Table 2) of glycosome matrix enzymes with high experimental evidence and/or targeting signal evidence of glycosome localization. We have demonstrated the feasibility of using the presence of PTS1 to identify additional glycosome matrix protein candidates in genomes and transcriptomes of kinetoplastid species, and we expect the number of testable transcriptomes and genomes to increase in number in the coming years [6]. Finally, for many glycosome-containing organisms with publicly available transcriptomes or genomes, we have performed an initial analysis of potential glycosome matrix proteins that may be present or absent in each particular genome (Figure 3). For instance, there are two hypoxanthine-guanine phosphoribosyltransferase paralogues in *T. brucei* and in the *Leishmania* species that we analyzed, both targeted to the glycosome. While we could only find one homologue in *T. cruzi* and some kinetoplastids, in others, only one of the two paralogues retained its PTS1, suggesting an expanded localization of this enzyme beyond the glycosome.

A bias of our approach is that our GCEC is derived from experimental studies in a few specific organisms, especially *T. brucei* proteome studies. If enzymes are compartmentalized into the glycosomes of monoxenous kinetoplastids, but not in the disease causing dixenous organisms, they are absent from our high-confidence lists. In the future it may be possible to interrogate several of the better-annotated monoxenous genomes such as *Leptomonas pyrrhocoris* [45] for PTS1-containing proteins to detect these potential glycosomal enzymes and even additional KEGG pathways that may be partially contained within kinetoplastid glycosomes.

Immediate and long-term future directions emerging from this research are apparent. In the short-term, with Table 2 proteins or future glycosome proteome studies of monoxenous organisms added to our GCEC dataset, we could re-train the PTS1-finding algorithm in an iterative approach to better understand this import signal. In tandem, we could utilize the genetically malleable *T. brucei* to better define appropriate PTS1 cutoff values for prediction of glycosome localization. For this approach, we would genetically tag and modify several PTS1s that score in the intermediate (~4–5.5) range for potential glycosome proteins, and microscopically visualize whether localization changes upon sequence modification. A similar approach was used to better define PTS1 in plants [29]. 

Longer term, several compelling research directions include characterizing the proteins of unknown function that reliably appear among the proteins purifying with glycosomes (Table 1 and Table 2). The fact that so many hypothetical proteins in the GCEC have no PTS1 or conserved PTS2 raises the possibility that perhaps they are membrane proteins that are not part of a glycosome compartmentalized metabolic pathway. Alternately, of course, they could simply possess yet-unidentified signals or piggyback on other glycosome proteins for entry. As a first step, their essentiality could be assessed fairly easily in a variety of kinetoplastids. It would also be intriguing to define a glycosome-specific version of PTS2, although multiple additional glycosome proteomic studies on a variety of kinetoplastids would likely be necessary to acquire the number of proteins needed to pull such a targeting signal out of the noise. Perhaps most globally applicable, one thing that we noted was the continued presence of a PTS1 on the glycolytic enzyme glyceraldehyde-3-phosphate dehydrogenase in one euglenoid species and glucose-6-phosphate isomerase in another, despite a widespread loss of PTS1 on the balance of the glycosomal glycolytic and gluconeogenesis pathway enzymes in the euglenoids. Interestingly, there is increasing evidence, particularly in yeast, of glycolytic proteins including glyceraldehyde-3-phosphate dehydrogenase being at least partially localized to the peroxisome [46]. A better understanding of the protein composition of glycosomes and the composition of the peroxisomes of closely related organisms may be important for understanding the purpose of compartmentalizing glycolytic enzymes among eukaryotes as a whole.

## 4. Materials and Methods

### 4.1. Scoring of Meta-Analysis

A repository of potential glycosomal proteins was created using tables and other data from glycosome purification and proteomic studies for three organisms: *T. brucei, T. cruzi,* and *Leishmania donavani*. Tables of likely glycosomal proteins in these studies were mined for those that were parts of enzymatic pathways or had no assigned function. The Peroxisomedb.org site’s Protein Families folder [19] was mined for our organisms of interest. Evaluation of protein localization in the TrypTag study initiated from an initial list of potentially glycosomal proteins from Dr. Samuel Dean, TrypTag co-developer, that we independently evaluated for the glycosome tagging pattern of oval-shaped organelles described in [24] using publicly available TrypTag images. Subsequently, TrypTag images of tagged versions of all of the remainder of Table S1 were individually analyzed the same way when available. 

Weighting of these studies was as follows: presence in [19] 1 point if yes, 0 points if no; presence in [22] 0.5 points for each life stage—procyclic and/or bloodstream forms—that it was observed in, 0 points if not found; presence in [20] 2 points if yes, 0 points if no; presence in [12] 2 points if yes, 0 points if no; presence in [11] 2 points if yes, 0 points if no, presence in [10] 2 points if yes, 0 points if no; glycosome localization pattern upon N-terminal tagging in TrypTag [21] 3 points if yes, 0 points if information was not available (tagging on the N-terminus was unsuccessful or not attempted), and −2 points if localization of signal from the N-terminus tagged protein was other than glycosomal (we utilized a lesser negative score for non-glycosomal localization because multiple non-relevant factors can lead to non-targeting of a tagged protein while a false positive is rare). As a C-terminal tagging could obscure a terminal peroxisome targeting signal 1, it was ignored. The cut-off score of 5 points was used, as it requires of a protein more than just inclusion in two *T. brucei* proteomic studies to reach this score. Sixty-four proteins made the cut-off value. These proteins were eventually decreased to 57 after combining *T. brucei*-specific duplications and the removal of glycosomal transporters from the list, as they were not deemed metabolic enzymes.

### 4.2. Categorization of Proteins into Metabolic Pathways

The proteins identified in our current analysis were categorized into groups based on the pathway in which they are involved (e.g., glycolysis/gluconeogenesis proteins). Kinetoplastid UniProt accession numbers were converted to KEGG identifiers using the Convert ID tool in the KEGG Mapper utility. The entries retrieved contained a KEGG reference ortholog and pathways ascribed to the given enzyme. Typically, the top most pathway retrieved was retained as the most broadly descriptive pathway for the search protein. As clusters of enzymes operating in more discrete sub-pathways were identified broad metabolic categories were replaced with specific sub-categories, such as the urea cycle in the degradation of multiple amino acids. The major category “Redox maintenance” is not a KEGG-derived pathway but was used to group the glutathione-based antioxidant cycle (glutathione metabolism) with the enzymes involved in detecting and metabolizing reactive oxygen species. BLASTP (Basic Local Alignment Search Tool for standard protein-protein search) search was used to identify orthologs of the hypothetical sequences but no orthologs were identified.

### 4.3. Identification of Orthologues in Other Organisms

Outside of the *Trypanosoma* and *Leishmania* genera, we identified additional sequenced genomes of glycosome-containing species and selected 21 for analysis. In addition, we also analyzed *Trypanosoma vivax* because of its presumably simpler lifestyle than *T. brucei*, *T. cruzi*, and *Leishmania tarentolae*, as its non-insect host is non-mammalian. Typically, paralogues were identical or nearly identical, and only one was selected for further analysis. We then performed analysis utilizing automated search functions and manual review to identify orthologues.

For the organisms *Blechomonas ayalai*, *Bodo saltans*, *Crithidia fasciculata*, *Endotrypanum monterogeii*, *Leishmania tarentolae*, *Leptomonas pyrrhocoris*, *Leptomonas seymouri*, *Paratrypanosoma confusum*, and *Trypanosoma vivax*, protein mining and homology searches of the *T. brucei* protein were done using TnBLAST at TriTryp [25] from August through October 2019, as this platform allowed for assurance of synteny. For the organisms *Angomonas deanei*, *Angomonas desouzai*, *Phytomonas serpens*, *Phytomonas* isolate EM1, *Phytomonas* isolate Hart 1, *Lotmaria passim*, *Strigomonas culicis*, *Strigomonas galati*, *Strigomonas oncopelti*, and *Herpetomonas muscarum*, the *T. brucei* orthologue of each GCEC protein was used to TBLASTN whole genome shotgun assembled contigs deposited in NCBI for the presence of Open Reading Frames (ORFs) with high sequence similarity. The best matched contigs were then analyzed using NCBI-ORF Finder to identify the high-similarity ORF. The ORFs were then aligned with the T. brucei standard using CLUSTAL-omega software to evaluate protein start and end positions and sequence similarity of all parts of the putative proteins. If partial and complete ORFs were present, the most complete ORF was selected. The best matched ORF was taken as the most likely orthologous proteins for that species/isolate. If the ORFs were fragmented into more than one contig, individual amino acid sequence fragments were manually joined to produce a single protein sequence. For *Trypanoplasma borreli*, the draft assembled genome was downloaded from the ENA database (accession SAMEA1948381) and searched as was performed for the Euglenoid species below.

In the cases of Diplonema and Euglenoid species, genome assembly has remained challenging [47,48,49]. However, transcriptomes are available for two Euglenoids. For *Eutreptiella gymnastica* (NCBI SRA accession SRX549022) and *Euglena gracilis* [50], sequences were retrieved from raw or previously assembled transcriptomic data. When necessary, we assembled the transcriptomic data using Trinity [51]. We then searched for homologues of the *T. brucei* 64 consensus glycosome proteins in these assembled genomes with NCBI’s BLAST+ [52]. For *Diplonema papillatum*, only the protein sequences identified in a previous study were used [15]. 

### 4.4. Development of Glycosome Targeting Signal 1 (PTS1) Algorithm

Our PTS1 score is derived directly from an additive log-odds score in each position. Site-specific frequencies q_i,j_ for each amino acid j at position i were calculated from the GCEC protein set, and corresponding negative frequencies p_i,j_ from the 35,640 other Uniprot proteins from *T. brucei*, *T. cruzi*, and *L. major*. Pseudocounts were added in computing the q_i,j_ to prevent zero valued frequencies. The PTS1 score is then ∑ ln(q_i,j_/p_i,j_) for the observed amino acids (j) at the last three C-terminal sites.

## Figures and Tables

**Table 1:** Glycosome Conserved Enzyme Collection (GCEC). Proteins from *Trypanosoma cruzi*, *Trypanosoma brucei*, and *Leishmania donovani* global studies of glycosome composition, evidence of localization of endogenous tagged proteins from the TrypTag project, and historical input of glycosome proteins in PeroxisomeDB culled from individual studies prior to 2010. Proteins are grouped by major KEGG (Kyoto Encyclopedia of Genes and Genomes) pathway inclusion. Presence of a targeting signal within the protein is indicated in the PTS1/PTS2 (Peroxisome Targeting Sequence 1/2) column (glycosome PTS1 or conserved peroxisome targeting signal 2, PTS2). It was not possible to assign a best KEGG reference orthologue for all proteins.

KEGG Reference Ortholog	Major KEGG Pathway	Name of Protein	PTS1/PTS2
**K18561**	Glycolysis	NADH-dependent fumarate reductase	PTS1
	Glycolysis	UDP-glc 4’-epimerase	PTS1
**K00850**	Glycolysis	ATP-dependent 6-phosphofructokinase, glycosomal	PTS1
**K00844**	Glycolysis/gluconeogenesis	Hexokinase 1	PTS2
**K01810**	Glycolysis/gluconeogenesis	Glucose-6-phosphate isomerase	PTS1
**K00134**	Glycolysis/gluconeogenesis	Glyceraldehyde 3-phosphate dehydrogenase	PTS1
**K01006**	Glycolysis/gluconeogenesis	Pyruvate phosphate dikinase	PTS1
**K00927**	Glycolysis/gluconeogenesis	Phosphoglycerate kinase	PTS2
**K01792**	Glycolysis/gluconeogenesis	Aldose 1-epimerase	PTS1
**K01623**	Glycolysis/gluconeogenesis	Fructose-bisphosphate aldolase	
**K01803**	Glycolysis/gluconeogenesis	Triose phosphate isomerase	
**K03841**	Gluconeogenesis	Fructose-1,6-bisphosphatase	PTS1
**K01610**	Gluconeogenesis	Phosphoenolpyruvate carboxykinase [ATP]	PTS1
**K00026**	Gluconeogenesis	Glycosomal malate dehydrogenase	PTS1
	Gluconeogenesis	UDP-glucose pyrophosphorylase	
**K00760**	Purine metabolism	Hypoxanthine-guanine phosphoribosyltransferase 1	PTS1
**K00760**	Purine metabolism	Hypoxanthine-guanine phosphoribosyltransferase 2	PTS1
**K00088**	Purine metabolism	Inosine-5’-monophosphate dehydrogenase	PTS1
**K00942**	Purine metabolism	Guanylate kinase	PTS1
**K00759**	Purine metabolism	Adenine phosphoribosyltransferase	PTS1
**K01490**	Purine metabolism	AMP deaminase	PTS1
**K00939**	Purine metabolism	Adenylate kinase	PTS1
**K00088**	Purine metabolism	Guanosine monophosphate reductase	PTS1
**K00036**	Pentose phosphate pathway	Glucose-6-phosphate 1-dehydrogenase (G6PD)	
**K00852**	Pentose phosphate pathway	Ribokinase	
**K01100**	Pentose phosphate pathway	Sedoheptulose-1,7-bisphosphatase	PTS1
**K01619**	Pentose phosphate pathway	Deoxyribose-phosphate aldolase	PTS1
**K00615**	Pentose phosphate pathway	Transketolase	PTS1
**K01057**	Pentose phosphate pathway	6-phosphogluconolactonase	
**K00864**	Glycerophospholipid metabolism	Glycerol kinase	PTS1
**K00803**	Glycerophospholipid metabolism	Alkyl-dihydroxyacetone phosphate synthase	PTS1
**K00649**	Glycerophospholipid metabolism	Dihydroxyacetonephosphate acyltransferase	PTS1
**K00006**	Glycerophospholipid metabolism	Glycerol-3-phosphate dehydrogenase (NAD(+))	PTS1
**K00022**	Fatty acid metabolism	Enoyl-CoA hydratase/Enoyl-CoA isomerase/3- hydroxyacyl-CoA dehydrogenase	PTS2
**K08766**	Fatty acid metabolism	Carnitine O-palmitoyltransferase	PTS1
**K11262**	Fatty acid metabolism	Acetyl-CoA carboxylase	
**K11207**	Redox maintenance	Trypanothione/tryparedoxin dependent peroxidase 2	
**K01833**	Redox maintenance	Trypanothione synthetase	
**K00103**	Redox maintenance	L-galactonolactone oxidase	PTS1
**K00869**	Terpenoid biosynthesis	Mevalonate kinase	PTS1
**K01823**	Terpenoid biosynthesis	Isopentenyl-diphosphate delta-isomerase (type II) (idi1)	
**K00031**	TCA cycle/glutathione metabolism	Isocitrate dehydrogenase	PTS1
**K01438**	Amino acid biosynthesis (arginine)	Acetylornithine deacetylase	PTS1
**K01107**	Insositol phosphate metabolism	Inositol polyphosphate 1-phosphatase	
**K13421**	Pyrimidine metabolism	Orotidine-5-phosphate decarboxylase/Orotate phosphoribosyltransferase	PTS1
**K15731**	RNA polymerase II C-terminal domain phosphatase	PTP1-interacting protein, 39 kDa/TFIIF-stimulated CTD phosphatase	PTS1
**K09829**	Steroid biosynthesis (ERG2)	C-8 sterol isomerase-like protein	PTS1
**K10703**	Long chain fatty acid synthesis	Protein tyrosine phosphatase	
**N/A**	N/A	Thymine-7-hydroxylase	PTS1
**N/A**	N/A	Hypothetical protein (Q580K0)	PTS1
**N/A**	N/A	Hypothetical protein (Q389Y7)	
**N/A**	N/A	Hypothetical protein (Q38C56)	
**N/A**	N/A	Hypothetical protein (Q386P8)	PTS1
**N/A**	N/A	Hypothetical protein(Q388J7)	
**N/A**	N/A	Hypothetical protein (Q38DM9)	
**N/A**	N/A	Hypothetical protein (Q383Q3)	
**N/A**	N/A	Hypothetical protein (Q38AC3)	

**Table 2:** Additional kinetoplastid proteins with both experimental evidence and a signal sequence indicating localization to the glycosome. Proteins are grouped by major KEGG pathway inclusion. It was not possible to assign a best KEGG orthologue for all proteins.

KEGG Reference Ortholog	Major KEGG Pathway	Name of Protein	PTS1/PTS2
**K00845**	Glycolysis	Glucokinase	PTS1
**K01809**	Glycolysis/gluconeogenesis	Phosphomannose isomerase	PTS1
**K17497**	Glycolysis/gluconeogenesis	Phosphomannomutase-like protein	PTS1
**K00849**	Glycolysis/gluconeogenesis	Galactokinase-like protein	PTS1
**K02564**	Glycolysis/gluconeogenesis	Glucosamine-6-phosphate isomerase	PTS1
**K00927**	Glycolysis/gluconeogenesis	Pas-domain containing phosphoglycerate kinase	PTS1
**K01443**	Glycolysis/gluconeogenesis	N-acetylglucosamine-6-phosphate deacetylase-like protein	PTS1
**K00863**	Glycolysis/gluconeogenesis	Dihydroxyacetone kinase 1-like	PTS1
**K01518**	Purine metabolism	Kinetoplastid-specific phospho-protein phosphatase	PTS1
**K00759**	Purine metabolism	Adenine phosphoribosyltransferase	PTS1
**K00853**	Pentose phosphate pathway	L-ribulokinase	PTS1
**K06128**	Glycerophospholipid metabolism	Lysophospholipase	PTS1
	Fatty acid metabolism	Acyl-CoA binding protein	PTS1
**K00311**	Fatty acid metabolism	Electron transfer flavoprotein-ubiquinone oxidoreductase	PTS1
**K13356**	Fatty acid metabolism	Fatty acyl- CoA reducatase	PTS1
**K00632**	Fatty acid metabolism	3-ketoacyl- CoA thiolase	PTS2
	Fatty acid epoxide hydrolase	Epoxide hydrolase	PTS1
**K04283**	Redox maintenance	Trypanothione-disulfide reductase	PTS1
**K11185**	Redox maintenance	Tryparedoxin peroxidase	PTS1
	Redox maintenance	Dj-1 family protein	PTS1
**K04564**	Redox maintenance	Iron superoxide dismutase	PTS1
**K04564**	Redox maintenance	Iron superoxide dismutase	PTS1
	Redox maintenance	2-oxoglutarate (2og) and Fe(II)-dependent oxygenase superfamily protein	PTS1
**K01940**	Urea cycle	Arginino-succinate synthase	PTS1
**K01438**	Urea cycle	Acetylornithine deacetylase-like	PTS1
**K01745**	Amino acid degradation	Histidine ammonia-lyase	PTS1
**K02614**	Amino acid degradation	Thioesterase-like superfamily	PTS1
	Peptide cleavage	Peptidase T	PTS2
	Protein cleavage	Carboxypeptidase M32	PTS2
**K02150**	pH regulation	V-ATPase, subunit E	PTS1
	Pyrophosphate and poly phosphate metabolism	Acidocalcisomal exopolyphosphatase	PTS1
**K02218**	Signal pathway regulation	Casein kinase I, isoform 2	PTS2
**K01676**	TCA cycle	Fumarate hydratase, class I (FHM)	PTS2
**K00972**	Amino and nucleotide sugar metabolism	UDP-N-acetylglucosamine pyrophosphorylase	PTS1
	N/A	Hypothetical protein (Q4DBW4)	PTS1
	N/A	Hypothetical protein (Q57TT5)	PTS1
	N/A	Hypothetical protein (Q381V8)	PTS1

## Supplementary Materials

The following are available online at https://www.mdpi.com/2076-0817/9/4/281/s1, Table S1. Complete list of putative enzymatic proteins with experimental evidence of glycosome localization from *Trypanosoma brucei*, *Trypanosoma cruzi*, and *Leishmania donovani* global studies of glycosome composition. *Leishmania major* is used as the representative Leishmania species in the listed orthologues of the dixenous human-disease causing typanosomatids. Studies represent those that define glycosome proteomes, provide evidence of localization of endogenous tagged proteins from the TypTag project, or that are reflected in the input of glycosome proteins in the Peroxisome database culled from individual studies prior to 2010. Table S2. Specific putative proteins and orthologue identities (Uniprot/TriTryp/NCBI contig numbers) used to determine glycosome targeting signal conservation across kinetoplastids, select euglinids and *Diplonema papillatum*. Contigs listed may be one of several that contain an identical or near-identical amino acid sequence for that specific protein. Different species or isolates of a genus are listed in separate columns. Each row represents one protein of our Glycosome Conserved Enzyme Collection. Black rectangles in the *D. papillatum* column represent proteins for which we did not attempt to find an orthologue. The *D. papillatum* entries that were used were those identified in [15]. A gene with its C-terminus at the extreme end of the contig may not be the complete gene; these are indicated by an asterisk when noted. Table S3. Kinetoplastid UniProt entries with PTS1 scores of 5 or higher. File S1. Code for PTS1 predictor. File S2. Fasta files containing *Trypanoplasma borreli*, *Euglena gracilis*, and *Eutreptiella gymnastica* mRNA coding sequence for orthologues of Glycosome Conserved Enzyme Collection proteins derived from publicly available genomic or transcriptomic data.
